# Are some teat disinfectant formulations more effective against specific bacteria isolated on teat skin than others?

**DOI:** 10.1186/s13028-019-0455-3

**Published:** 2019-04-25

**Authors:** Sarah Rose Fitzpatrick, Mary Garvey, Jim Flynn, Kieran Jordan, David Gleeson

**Affiliations:** 1 Teagasc, Animal & Grassland Research and Innovation Centre, Moorepark, Fermoy, County Cork, Ireland; 2 Cellular Health and Toxicology Research Group, Institute of Technology Sligo, County Sligo, Ireland; 3 Teagasc Food Research Centre, Moorepark, Fermoy, County Cork, Ireland

**Keywords:** Dairy cows, Dairy hygiene, Swabs, Teat bacterial load, Teat disinfection

## Abstract

The use of pre- and post-milking teat disinfectants can reduce teat bacterial load and aid in the collection of high-quality milk. The objective of this study was to compare the reduction in bacteria populations on teat skin after the application of different commercial teat disinfectant products. Ten teat disinfectant products were applied to the teats of 10 Holstein–Friesian cows. One cow received one teat disinfectant product at each sampling point before cluster application for milking. A composite swab sample was taken of the 4 teats of each cow before and after teat disinfectant application. Swab samples were placed on three different selective agars to enumerate bacterial counts of staphylococcal, streptococcal and coliforms isolates on teat skin. Staphylococcal isolates were the most prominent bacterial group recovered on teat swabs (49%), followed by streptococcal (36%) and coliform (15%) isolates before the application of disinfectant. The average bacterial reductions on teat skin were shown to be 76%, 73% and 60% for staphylococcal, streptococcal and coliform isolates, respectively. All of the teat disinfectant products tested reduced teat bacterial load for all three bacterial groups. Product 4 containing 0.6% w/w diamine was the most effective against bacterial populations of staphylococcal and streptococcal isolates on teat skin with a reduction of 90% and 94%, respectively. Whereas product 10, which contained 0.5% w/w iodine, resulted in the highest reduction in coliforms on teat skin with a reduction of 91%. Results from this study suggest that specific bacterial population loads on teats can be reduced using different teat disinfectant formulations.

## Findings

The teat orifice is the first line of defence from the invasion of mastitis pathogens into the teat canal and mammary glands. High bacterial contamination may increase the chances of more bacteria entering the teat orifice and causing infection [1]. The removal of bacteria pre- and post-milking can lower the occurrence of new intramammary infections (IMI’s) [2, 3]. Therefore, determining the efficacy of teat disinfectant products against bacteria naturally present on teat skin is important. Teat swabbing has been used to determine the effect of pre-milking teat preparation treatments [1, 2, 4–6], enumerate and identify bacteria present on teat skin surface [7–11]. Previous studies focused on evaluating pre-milking teat cleaning procedures. Whereas, this study will determine the impact of 10 pre- and post-milking teat disinfectant products, with different ingredients of varying concentration, on the reduction of teat skin bacterial load.

This study was approved by the Teagasc Animal Ethics Committee (ref. TAEC168-2017). The BS EN 1656 is a European standard which is used to test chemical disinfectants used in the veterinary area against bacteria recommended by the standard. To meet the requirements of this standard for teat disinfectants, the product must demonstrate at least a 10^5^ log reduction (99.999% reduction) within 5 min against *Staphylococcus aureus* (ATCC^®^ 6538™), *Streptococcus uberis* (ATCC^®^ 19436™) and *Escherichia coli* (ATCC^®^ 10536™). Before the disinfectant products were applied to cow’s teats, each product was tested using the BS EN 1656. Ten disinfectant products (Table 1) were applied to the teats of 10 Holstein–Friesian cows. All teat disinfectant products were ready-to-use (RTU) while one product (product 7) was mixed with an activator before use, according to manufacturer’s recommendations, and was considered a RTU product by the manufacturer. The disinfectant products were suitable for both pre- and post-milking teat disinfection, with the exception of products 7 and 10, which were recommended for use only for post-milking disinfection. The cows were housed, in one group, indoors, on matted cubicle beds dressed with ground limestone daily to maintain a dry bed.

**Table 1:** Test teat disinfectant product code and active ingredient, as declared by the manufacturer on product label

Code	Product	Ingredient	Manufacturer/supplier
1	Lacto-cel^a^	2.4% w/w lactic acid	Biocel Ltd.
2	Duogold^a^	2% w/w lactic acid and 0.3% w/w chlorhexidine gluconate	Gold Assure
3	Arkshield^a^	5% w/w lactic acid and 0.3% w/w chlorhexidine	Ark Farm Innovations Ltd.
4	Super cow teat foam^a^	0.6% w/w diamine	Milk Solutions Ltd.
5	Sensodip 50^a^	0.5% w/w chlorhexidine	GEA Farm Technologies Ltd.
6	PureChem chlorhexidine^a^	0.29% w/w chlorhexidine	Central Chemical Supplies Ltd.
7	Kenomix^b^	0.0157% w/w chlorine dioxide	CID Lines N.V.
8	Lanodip pre-post^a^	0.29% w/w iodine and 0.8% w/w lactic acid	Kilco International Ltd.
9	Hypred quick spray^a^	2% w/w lactic and 0.1% w/w salicylic acid	Grassland Agro Ltd.
10	Maxidine RTU^b^	0.5% w/w iodine	Biocel Ltd.

^a^Pre- and post-milking application

^b^Post-milking application only

Before sampling, swabs were moistened [4] in sterile trypticase soy broth (TSB) (Merck Millipore, Ireland) to aid in the collection of bacteria from the teat skin before and after teat disinfectant application. Over 10 milkings (AM and PM; 5 days), teat disinfectant products were applied to the teats of 10 cows (10 replicates per product). Every cow received each product once over the duration of the trial, with a different product applied at each milking. Before (PRE) the application of a test teat disinfectant, a composite teat skin swab was collected from all 4 teats. For PRE swab samples, swabs were drawn across the teat orifice and down the side of each teat avoiding contact with the udder hair and cows flank [2, 10]. All teats of the cow were then immersed in a test teat disinfectant using a teat dip cup. The teat disinfectant was then left on the teat skin for up to 1 min. Next, teats were dry wiped with a single-use paper towel. A pilot study, where the inclusion of a dry wipe was compared to no dry wipe after application of teat disinfectant products, showed that there was no difference in the reduction of bacterial load on teat skin. Following this, composite swab samples were collected (POST) from all 4 teats in a similar manner to that for the PRE samples. However, POST samples were collected on the opposite side of the teats. Immediately after sampling, swabs were placed into individual sterile bottles containing 10 mL of sterile TSB and neutraliser (30 g/L polysorbate 80 and 3 g/L l-α-phosphatidylcholine from egg yolk) and placed in storage at − 20 °C [10], within 1 h of sampling, for 7–14 days before undergoing laboratory analysis. A previous study demonstrated that storage of skin swab samples at different temperatures did not affect the abundance or diversity of bacterial population [12]. A total of 200 teat swab samples were collected during the trial. For the bacterial counts, maximum recovery diluent was used to make 1:100 dilutions in sterile tubes for the PRE samples. The POST sample was used undiluted. The samples were subsequently plated, in triplicate, onto 3 separate agars; Baird parker agar (Merck Millipore, Ireland) for staphylococcal isolates, modified Edwards agar (Sigma-Aldrich, Ireland) with 5% sterile blood for streptococcal isolates and MacConkey agar (Merck Millipore, Ireland) for coliform isolates [1]. Following incubation at 37 °C for 24 h, microbial counts for each bacterial group were manually counted. Bacterial species within each isolate group were not defined.

Bacterial counts (cfu/mL) were transformed to base-10 logarithm for analysis. Statistical analysis was performed using SAS version 9.4 [13]. Reduction in bacteria of teat skin was calculated as the difference between the Log_10_ values of PRE and POST. PROC GLIMMIX was used to perform multiple pair-wise comparisons. The LSMEANS statement in PROC GLIMMIX was used to differentiate statistical differences. Residual checks were made to ensure assumptions of analysis were met. The reductions for the bacterial isolate groups tested were analysed using 3 models (one for each bacterial group). This model included the reduction as a dependent variable and product, day and time as independent variables. The equation for all models was; $Reduction = Product + Day + Time + Day \times Product + Time \times Product$, where reduction was the base-10 logarithm of the cfu/mL unit of interest + 1, product was the products tested, day was the date of sampling and time refers to the milking the swab was collected (AM or PM). The cow was the experimental unit.

In this study, all 10 disinfectant products were tested using the BS EN 1656 protocol. All products achieved a log reduction ranging from 5.31 to 5.96 within 5 min of treatment time for the 3 recommended bacteria. Therefore, indicating that the disinfectant products provide sufficient levels of inactivation and meet the requirements of the BS EN 1656.

Overall, day had a significant effect on the bacterial numbers on teats within the study but there was no day by product effect for all three bacterial groups (P > 0.05). Furthermore, the time of collection (AM or PM milking) had no significant effect on the reduction of bacterial numbers (P > 0.09), but bacterial counts were higher for all swabs collected in the AM milking in comparison to swabs collected in the PM milking. The difference between bacterial counts for time of sampling may be due to the different time intervals between milkings (i.e. 7 h between AM and PM milking and 17 h between PM and AM milking). In natural exposure studies, day and time of collection may impact bacterial load on teat skin due to factors such as; management and environment, which can cause a fluctuation of bacterial load on skin surface [4, 5, 14, 15].

**Fig. 1:**
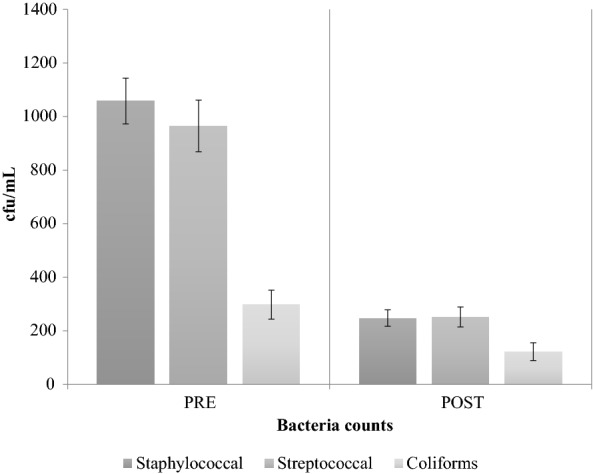
Overall means for staphylococcal, streptococcal and coliform isolate counts (cfu/mL) on teat swab samples before (PRE) and after (POST) the application of test teat disinfectant products. Error bars indicate SEM

Staphylococcal isolates were the most prominent bacterial group recovered on teat swabs (49%), followed by streptococcal (36%) and coliform (15%) isolates. This was similar to previous studies where staphylococcal species were more abundant than streptococcal species [1, 2]. All teat disinfectant products used in the study reduced bacterial load on teat skin. The overall reduction in bacterial counts was significant (P < 0.05) across all treatments for staphylococcal, streptococcal and coliform isolates (Fig. 1). These results agree with previous studies which showed that teat disinfection reduced bacterial load on teat skin [1, 2, 5], but the reduction of bacterial load was slightly lower in this study in comparison to other studies [1, 4].

The results for each bacterial isolate group can be observed in Table 2. For staphylococcal isolates, average bacterial reduction on teat skin was 76%, (range; 56% - 90%). Products containing 0.6% diamine (product 4) and 0.5% chlorhexidine (product 5) were the most effective in reducing the bacterial load on teat skin, with both products giving a bacterial reduction of 90%. The average bacterial reduction on teat skin for streptococcal isolates was 73% (range: 59%–93%). A product containing 0.6% diamine (product 4) was the most effective in reducing the bacterial load on teat skin, achieving a reduction of 94%. Coliform bacterial load on teat skin was reduced, on average, by 60% (range: 20%–88%). A product containing 0.5% w/w iodine (product 10) was the most effective and achieved a reduction of 91%. This agrees with other studies where iodine has been shown to be effective against a wide range of staphylococcal [2, 5, 16] streptococcal and coliform species [1, 2, 5, 6].

**Table 2:** The PRE and POST cfu/mL values and log and cfu/mL reduction of staphylococcal, streptococcal and coliform isolates on teat skin swabs

Product	Staphylococcal				Streptococcal				Coliforms			
	PRE cfu/mL	POST cfu/mL	cfu/mL reduction	Log reduction	PRE cfu/mL	POST cfu/mL	cfu/mL reduction	Log reduction	PRE cfu/mL	POST cfu/mL	cfu/mL reduction	Log reduction
1	1215	255	959	0.75^b^	1203	259	944	0.66^ab^	193	55	138	1.56^a^
2	1055	310	744	0.76^b^	987	233	754	0.65^ab^	385	263	121	1.22^a^
3	1184	303	800	1.04^ab^	1151	449	702	0.63^ab^	251	121	130	1.26^a^
4	1280	125	1155	2.09^a^	1171	74	1097	1.91^a^	494	115	379	1.60^a^
5	985	101	884	1.16^ab^	923	155	767	1.05^ab^	177	77	100	1.71^a^
6	950	185	765	0.91^ab^	830	340	490	0.74^ab^	274	132	142	0.83^a^
7	953	269	684	0.92^ab^	883	336	547	1.01^ab^	290	35	255	1.28^a^
8	936	251	685	0.71^b^	652	111	541	0.78^ab^	347	277	70	1.24^a^
9	949	414	535	0.40^b^	710	265	445	0.24^b^	319	125	193	1.58^a^
10	1076	263	814	0.42^b^	1140	295	845	0.82^ab^	250	22	228	1.77^a^

*PRE* teat skin swab sample before teat disinfectant application, *POST* teat skin swab sample after teat disinfectant application

Product: 1 = Lacto-cel, 2 = Duogold, 3 = Arkshield, 4 = Super Cow Teat Foam, 5 = Sensodip 50, 6 = PureChem Chlorhexidine, 7 = Kenomix, 8 = Lanodip Pre-Post, 9 = Hypred Quick Spray, 10 = Maxidine RTU. The log reduction means of staphylococcal, streptococcal and coliform isolates with different letters are statistically significant (P < 0.05)

A limitation of the study was the lower log reduction obtained using teat swabbing in comparison to the laboratory method, BS EN 1656. This may have been influenced by the low initial level of bacterial isolates present on teat skin prior to the application of disinfectant products. Challenging the teat skin surface with a known concentration of a specific bacterial strain, rather than depending on the natural bacteria present, may help to ensure initial levels of bacteria on teat skin to reflect log reductions required in the BS EN 1656. Furthermore, the time period in which the teat disinfectant products were left on the teat skin (1 min) may not have been long enough to make a comparison with the BS EN 1656 as this protocol requires a treatment time of 5 min. In addition, when swabbing the teat before and after teat disinfection, it was considered important to include the teat orifice in both sample collections as microbial colonisation of the teat canal and orifice can serve as a reservoir for the development of new IMIs during lactation [17].

In conclusion, all teat disinfectant products used in this study can reduce the bacterial load on teat skin of dairy cows. Additionally, a variation in sensitivity and resistance to active ingredients was observed across the bacteria isolates tested. Furthermore, longer natural exposure trials should be undertaken to evaluate the efficacy of the test teat disinfectants ability to reduce new IMIs.

## Abbreviations

IMI: intramammary infection
POST: swab sample collected 1 min after teat disinfection
PRE: swab sample collected before teat disinfection
SEM: standard error mean
TSB: tryptic soy broth
